# Eicosapentaenoic Acid Suppresses Tumor Growth and Enhances Chemosensitivity via AKT/mTOR Signaling in Uterine Serous Carcinoma

**DOI:** 10.3390/cancers18071120

**Published:** 2026-03-31

**Authors:** Haomeng Zhang, Weimin Kong, Xiaochang Shen, Shuning Chen, Glenn Boyles, Chelsey Vranes, Miller Singleton, Alexandra Diggs, Chunxiao Zhou, Victoria L. Bae-Jump

**Affiliations:** 1 Department of Gynecology, Beijing Obstetrics and Gynecology Hospital, Capital Medical University, Beijing Maternal and Child Health Care Hospital, Beijing 100026, China; 2 Division of Gynecologic Oncology, University of North Carolina at Chapel Hill, Chapel Hill, NC 27599, USA; 3 Lineberger Comprehensive Cancer Center, University of North Carolina at Chapel Hill, Chapel Hill, NC 27599, USA

**Keywords:** uterine serous carcinoma, eicosapentaenoic acid (EPA), apoptosis, synergy, invasion

## Abstract

Background/Objectives: Uterine serous carcinoma (USC) is an aggressive subtype of endometrial cancer characterized by high recurrence rates and poor response to conventional therapies, resulting in unfavorable clinical outcomes. Eicosapentaenoic acid (EPA), an omega-3 polyunsaturated fatty acid, has demonstrated anti-cancer activity in multiple malignancies. Methods: This study used two USC cell lines, ARK1 and SPEC2, to evaluate the effects of EPA on cell proliferation, invasion, cell cycle profile, stress response, and apoptosis. The potential synergistic effects of EPA combined with carboplatin were also examined. Western blotting was used to examine EPA’s effects on downstream pathways related to cellular stress, inflammation, and epithelial–mesenchymal transition (EMT). Results: EPA treatment markedly reduced cell proliferation and colony formation, with IC_50_ values of 28.96 µM for ARK-1 cells and 14.96 µM for SPEC-2 cells compared with control groups. It also induced G1 phase cell cycle arrest, increased cellular stress, triggered caspase-dependent apoptotic cell death, and suppressed invasive capacity. Moreover, EPA effectively counteracted TNF-α-stimulated upregulation of COX-2 and phosphorylated NF-κB. The combined treatment with EPA and carboplatin resulted in synergistic inhibition of cell viability and migration. Western blotting analysis showed that EPA attenuates the NF-κB and AKT/mTOR signaling pathways, promotes the expression of cellular stress-related proteins, and inhibits the expression of EMT-related proteins in both cell lines. Conclusions: EPA exhibits potent anti-tumor activity against USC cells and enhances the efficacy of carboplatin. These data indicate that EPA has potential as a low-toxicity, multi-target adjuvant treatment for USC, necessitating additional pre-clinical and clinical investigation.

## Simple Summary

Uterine serous carcinoma (USC) is a highly aggressive subtype of endometrial cancer. Eicosapentaenoic acid (EPA) has been reported to exhibit anti-cancer properties across various malignancies. The aim of our study was to assess the therapeutic potential in the context of USC. We confirmed that EPA treatment reduced cell growth, colony formation, and invasive capabilities in the ARK1 and SPEC-2 cell lines, while inducing cell cycle arrest in the G1 phase, attenuating AKT/mTOR signaling pathways and inducing caspase-dependent apoptotic cell death. Moreover, EPA effectively counteracted TNF-α-stimulated upregulation of COX-2 and phosphorylated NF-κB. The combined treatment with EPA and carboplatin resulted in synergistic inhibition of cell viability and migration. Therefore, EPA has potential as a low-toxicity, multi-target adjuvant treatment for USC, necessitating additional pre-clinical and clinical investigation.

## 1. Introduction

Uterine serous carcinoma (USC) is a highly aggressive histological variant of endometrial carcinoma, representing approximately 10% of all endometrial cancer cases [1,2,3]. It is characterized by an aggressive clinical course, with a tendency for early extrauterine dissemination and high rates of recurrence, even when the tumor is confined to the uterus at diagnosis. Consequently, USC has a significantly worse prognosis relative to stage-matched endometrioid endometrial carcinoma, accounting for nearly 40–60% of endometrial cancer–related mortality [1,4,5]. Current treatments use a multimodal strategy that may include surgery, radiation, chemotherapy, and, in some instances, targeted therapies [5,6]. Despite these treatment options, the five-year overall survival rate for USC remains low, ranging from only 18% to 27%. These poor survival outcomes, coupled with the aggressive biological behavior of USC, underscore the urgent need for more effective therapeutic approaches for patients affected by this disease [5,6,7].

**Figure 1:**
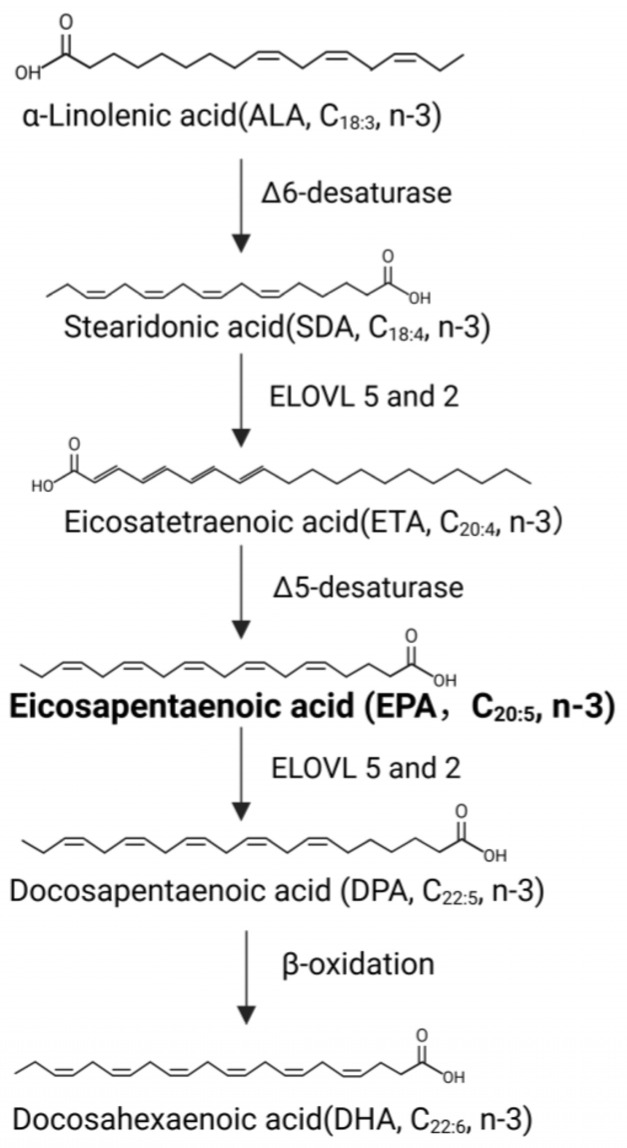
Biosynthesis and chemical structures of EPA and other PUFAs. The synthesis of EPA, (C20:5, n−3) begins with α-linolenic acid (ALA, C18:3, n−3), which undergoes Δ6-desaturation to form stearidonic acid (SDA, C18:4, n−3). SDA is subsequently elongated by elongases (ELOVL5/2) to eicosatetraenoic acid (ETA, C20:4, n−3), followed by Δ5-desaturation to generate EPA. EPA is further elongated to docosapentaenoic acid (DPA, C22:5, n−3). Subsequent Δ6-desaturation and peroxisomal β-oxidation yield docosahexaenoic acid (DHA, C22:6, n−3).

Eicosapentaenoic acid (EPA) is a 20-carbon omega-3 polyunsaturated fatty acid (PUFA) with five cis double bonds, found abundantly in fish and other marine sources (Figure 1) [8,9]. As an essential nutrient, EPA regulates lipid metabolism and maintains membrane fluidity, partly through its conversion into bioactive lipid mediators such as resolvins and other specialized pro-resolving mediators, which support cardiovascular health, metabolic benefits, and mental well-being [8,9,10,11]. Although epidemiological and clinical investigations have produced conflicting results regarding the impact of dietary EPA on preventing and treating cancer, accumulating preclinical evidence indicates that EPA exerts anti-tumorigenic effects across a range of malignancies, including breast, colorectal, ovarian, and pancreatic cancers, through multiple mechanisms, including induction of cell cycle arrest and apoptosis, causing immunogenic cell death, suppression of inflammation and angiogenesis, enhancing the efficacy of chemotherapeutic agents, alternating membrane composition, and modulating key oncogenic signaling pathways [12,13,14,15]. We previously reported that docosahexaenoic acid (DHA), a 22-carbon omega-3 PUFA, exerts strong anti-proliferative and anti-invasive activities in our transgenic mouse model of serous epithelial ovarian cancer, indicating that long-chain omega-3 PUFAs may hold therapeutic potential in serous-type gynecologic cancers [16].

Given the current lack of effective treatment strategies for advanced and recurrent USC and the potential anti-tumorigenic activity of EPA against serous histologies, it is necessary to investigate the potential impact of EPA on cell viability, invasiveness, and synergistic effects with and without chemotherapy drugs in USC cells. Therefore, this study aims to comprehensively evaluate the inhibitory effects of EPA on cell growth and invasion, assess its potential synergistic effect with carboplatin, and explore the molecular mechanism of its anti-cancer activity in USC cells.

## 2. Materials and Methods

### 2.1. Cell Culture

The ARK-1 and SPEC2 cell lines were used for all subsequent experiments. ARK-1, obtained from Dr. Santin (Yale University School of Medicine), which retains functional PTEN with relatively moderate PI3K/AKT pathway activation, was cultured in RPMI 1640 medium supplemented with 10% fetal bovine serum (FBS) (Phoenix Scientific, St. Joseph, MO, USA), 100 units/mL penicillin–streptomycin, and 1% L-glutamine (Thermo Fisher Scientific, Waltham, MA, USA). SPEC2 which is characterized by PTEN loss and constitutively high AKT activity, generously provided by Dr. Kauffman (University of North Carolina at Chapel Hill, Chapel Hill, NC, USA), was maintained in Dulbecco’s Modified Eagle Medium/Nutrient Mixture F-12 (DMEM/F12) supplemented with 10% FBS. Both cell lines harbor TP53 mutations. Both cell lines were passaged every 2–3 days and incubated at 37 °C in a humidified atmosphere with 5% CO_2_.

### 2.2. Reagents

Analytical grade EPA (purity ≥ 99%) was purchased from Cayman Chemical (Ann Arbor, MI, USA). Carboplatin (Cat. No.: HY-17393) was obtained from MedChemExpress (Monmouth Junction, NJ, USA). 3-(4,5-Dimethylthiazol-2-yl)-2,5-diphenyltetrazolium bromide (MTT), dimethyl sulfoxide (DMSO) and other chemical reagents were provided by Thermo Fisher Scientific (Waltham, MA, USA) and Sigma-Aldrich (St. Louis, MO, USA). All antibodies were acquired from ABclonal (Woburn, MA, USA) and Cell Signaling Technology (Beverly, MA, USA). The ELISA substrates Ac-DEVD AMC, Ac-IETD-AFC, and Ac-LEHD-AMC were obtained from AAT Bioquest (Pleasanton, CA, USA). Propidium iodide (PI) was purchased from Sigma-Aldrich (St. Louis, MO, USA).

### 2.3. Cell Proliferation Assay

The methylthiazolyldiphenyl-tetrazolium bromide (MTT) assay was performed to evaluate cell proliferation. A total of 4000 ARK-1 and 8000 SPEC2 cells were seeded into 96-well plates (GenClone, San Diego, CA, USA) and cultured for 24 h. The cells were then exposed to EPA at a concentrations of 0, 0.1, 1, 10, 25, 50, 100, 250, 500 µM for around 72 h. A total of 5 µL of 5 mg/mL MTT solution was added to the cells which were then incubated for 1 h at 37 °C in the dark. After removal of the supernatant, 100 µL DMSO was added equally into each well [17]. The absorbance value was detected at 562 nm, using a Thermo microplate reader (Cleveland, OH, USA). The IC_50_ (half-maximal inhibitory concentration) values were calculated using the AAT Bioquest online calculator.

### 2.4. Colony Assay

Cells were plated in 6-well plates at a density of 400–600 cells per well and exposed to different EPA concentrations (1, 50, 100 µM for ARK-1, 1, 10, 50 µM for SPEC2) for 36 h. After treatment, the cells were cultured in fresh culture medium for 12–14 days. The colonies were stained with crystal violet solution for 45 min and then rinsed with tap water for 30 min. After the plates dried out, the colonies that had more than 50 cells were counted under the microscope [18].

### 2.5. Western Blotting Assay

After exposure to EPA (1, 50, 100 µM for ARK-1 and 1, 10, 50 µM for SPEC2) for 6–24 h, the ARK-1 and SPEC2 cells were lysed in RIPA buffer supplemented with a phosphatase and protease inhibitor cocktail. Total protein concentration was measured by BCA assay (Thermo Fisher Scientific, Cleveland, OH, USA). Equal amounts of the protein samples were mixed with loading buffer and boiled for 4 min. After separation by SDS-polyacrylamide gel electrophoresis, the proteins were transferred to an equilibrated PVDF membrane (Bio-Rad, Hercules, CA, USA). Membranes were blocked in 5% nonfat powdered milk for 1 h at room temperature and covered with the primary antibody overnight in a cold room. On the second day, the membranes were incubated with a rabbit or mouse-specific secondary antibody. The target protein bands were developed by Western Lightning Plus-ECL (PerkinElmer, Waltham, MA, USA) on the ChemiDoc Image System with Image Lab software (version 6.1, Bio-Rad, Hercules, CA, USA) [16]. All Western blotting experiments were repeated three times.

### 2.6. Cell Cycle Analysis

The cells were seeded into 6-well plates overnight and subsequently exposed to EPA (1, 50, 100 µM for ARK-1 and 1, 10, 50 µM for SPEC2) or vehicle for 24 h. Cells were then harvested by 0.25% non-EDTA trypsin and collected, then fixed with cold methanol and stored on the ice for 20 min. After centrifugation, the cells were washed with ice-cold PBS twice then stained with PI in the incubator for 30 min in the dark. Samples were measured by Cellometer (Nexcelom, Lawrence, MA, USA) immediately, and analyzed by FCS Express software (version 7.28, De Novo Software, Glendale, CA, USA) [19].

### 2.7. Reactive Oxygen Species (ROS) Assay

To detect ROS production, the ARK-1 and SPEC2 cell lines were grown in 96-well plates for 24 h and then treated with the EPA (1, 50, 100 µM for ARK-1 and 1, 10, 50 µM for SPEC2) for 4 h. Phenol red-free medium with the oxidation-sensitive probe 2’,7’-dichlorofluorescin diacetate (DCFH-CA) was added to each well for 30 min. The nonfluorescent DCF is converted mainly by ROS to a fluorescent derivative, which was then measured at 485 nm excitation and 525 nm emission wavelengths [20].

### 2.8. Mitochondrial Membrane Potential Analysis

The ARK-1 and SPEC2 cells were treated with different concentrations of EPA (1, 50, 100 µM for ARK-1 and 1, 10, 50 µM for SPEC2) for 3 h and then stained with 2 µM JC-1 for 30 min avoiding light. The cells were rinsed twice with PBS and immediately measured at wavelengths Ex/Em 485/535 nm by a Thermo microplate reader [21].

### 2.9. Cleaved Caspase 3, 8, and 9 ELISA Assays

We performed ELISA detection of lysed caspase 3, 8, and 9 using our previously published method [22]. The cell lines were exposed to various concentrations of EPA (1, 50, 100 µM for ARK-1 and 1, 10, 50 µM for SPEC2) for 4 h to evaluate apoptosis. Following treatment, the supernatant was aspirated, and the wells were washed twice with cold PBS. Each well then received 150 µL of lysis buffer. The resulting cell lysates were incubated with the appropriate cleaved caspase substrates and DTT in reaction buffer for 30 min in the dark. The mixtures were transferred to a black, clear-bottom 96-well plate, and fluorescence was measured using a Thermo microplate reader. Cleaved caspase-3 and caspase-9 were detected at Ex/Em 341/441 nm, and cleaved caspase-8 at Ex/Em 376/482 nm.

### 2.10. Adhesion Assay

Adhesion assay was performed using laminin-1 coated 96-well plates [22]. The plates were incubated with blocking buffer which contained 5% BSA in PBS for 30 min. The ARK-1 and SPEC2 cell lines (2 × 10^5^ cells/mL) were exposed to different concentrations of EPA in culture plates and incubated with the lid open for 90 min. A total of 100 µL 5% glutaraldehyde was added to the plates for 30 min after EPA treatment. After staining with 100 µL of crystal violet solution, the plates were then solubilized with 100 µL acetic acid. Finally, cell adhesion was quantified by measuring the absorbance at 562 nm, and results were expressed as the percentage of adherent cells in each well.

### 2.11. Wound Healing Assay

Both cell lines were inoculated into 6-well plates with 90% confluence by the following day. Three linear wounds were scratched across the monolayer by a 200 µL pipette tip. After the cells were washed with PBS and cultured with fresh medium, photographs were taken at 0 h and 14 h after EPA treatment (1, 50, 100 µM for ARK-1 and 1, 10, 50 µM for SPEC2). Wound closure was monitored and imaged under a microscope, and the migration ability was quantified by calculating the scratch-healing rate [23].

### 2.12. Statistical Analysis

All statistical analyses and figure generation were performed using GraphPad Prism 10 (San Diego, CA, USA). Statistical differences between groups were evaluated using either Student’s *T*-test or one-way ANOVA, depending on the experimental design. All experiments were repeated three times, and the data expressed as mean ±  standard deviation (SD). A *p*-value < 0.05 was considered statistically significant. Statistical significance is indicated as * *p* < 0.05 and ** *p* < 0.01.

## 3. Result

### 3.1. EPA Inhibits Cell Proliferation in USC Cells

**Figure 2:**
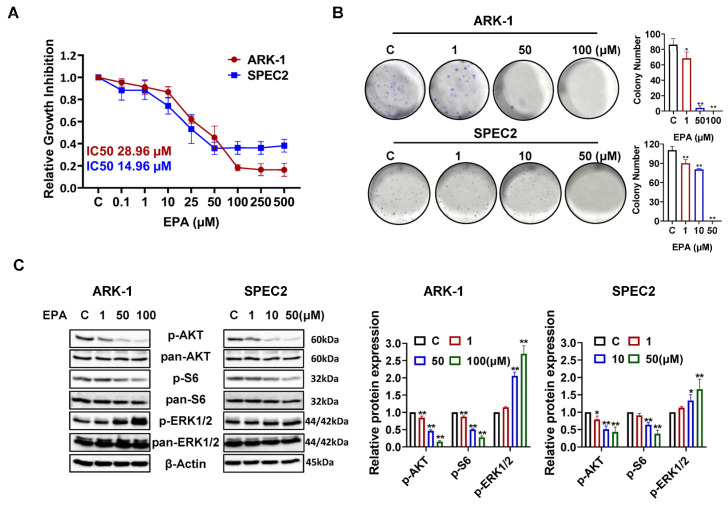
EPA inhibited USC cell proliferation. Both ARK-1 and SPEC2 were cultured at 4000–8000 cells/well, and treated with different concentrations of EPA for 72 h. MTT results showed that EPA inhibited cell proliferation in the ARK-1 and SPEC2 cell lines (**A**). ARK-1 and SPEC2 were seeded into 6-well plates at 400 cells/well, and incubated with EPA (1, 50, 100 μM for ARK-1, and 1, 10, 50 μM for SPEC2) for 36 h. The plates were then cultured for 12–14 days. EPA markedly inhibited colony formation in ARK-1 and SPEC2 cells in a dose-dependent manner. In ARK-1, 1 μM EPA reduced colony formation by 20.54% (*p* = 0.0175), 50 μM by 95.35% (*p* < 0.0001), and 100 μM by 100% (*p* < 0.0001). In SPEC2, 1 μM EPA reduced colonies by 18.43% (*p* = 0.0011), 10 μM by 27.19% (*p* < 0.0001), and 50 μM by 100% (*p* < 0.0001) (**B**). Both cell lines were treated with EPA (1, 50, 100 μM for ARK-1 and 1, 10, 50 μM for SPEC) for 24 h. Western blotting results revealed that the EPA decreased phosphorylated (p)-AKT and p-S6 expression level and increased p-ERK1/2 expression activity after treatment of ARK-1 and SPEC-2 cells for 24 h (**C**). For uncropped Western blot figures see Figure S1. * *p* < 0.05, ** *p* < 0.01 compared with control.

To explore the impact of EPA on USC cell proliferation, the ARK-1 and SPEC2 cells were seeded into 96-well plates and treated with different doses of EPA (0, 0.1, 1, 10, 25, 50, 100, 250, 500 µM) for 72 h. The MTT assay results demonstrated that EPA significantly reduced cell proliferation, with an IC_50_ value of 28.96 µM for ARK-1 cells and 14.96 µM for SPEC2 cells (Figure 2A). Since colony formation assay is widely considered a strong predictor of cell responsiveness to anti-cancer compounds [24], we then evaluated the effect of EPA on the colony formation ability of the USC cell lines. After treatment of ARK-1 and SPEC2 cells with EPA at concentrations ranging from 1 to 100 µM for 36 h, the cells were cultured for another 12–14 days, and fresh medium was changed every three days. EPA effectively inhibited the colony-forming ability of USC cells. 50 µM EPA decreased colony formation by 95.35% in ARK-1 cells (*p* < 0.01) and by 100% in SPEC2 cells (*p* < 0.01) (Figure 2B). These results suggest that EPA can effectively inhibit cell growth in USC.

Given that ω-3 PUFAs can interact with the AKT/mTOR and MAPK signaling pathways in cancer cells, we then investigated the impact of EPA on these pathways in USC cells using Western blotting analysis. After 24 h of EPA treatment, there was a concentration-dependent decrease in the expression levels of phosphorylated AKT and phosphorylated S6, along with an increase in the expression of phosphorylated ERK1/2 (Figure 2C), indicating that EPA-mediated growth inhibition involves regulation of both the PI3K/AKT/mTOR and MAPK/ERK signaling cascades.

### 3.2. EPA Induces G1 Phase Cell Cycle Arrest in USC Cells

**Figure 3:**
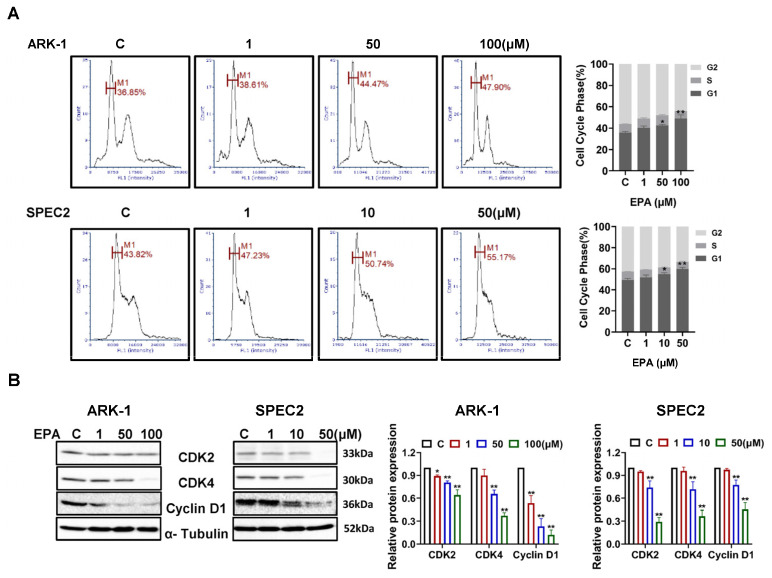
EPA caused G1 cell cycle arrest in USC cells. ARK-1 was treated with 1, 50 and 100 μM EPA, and SPEC-2 was treated with 1, 10, 50 μM EPA for 24 h. Cell cycle profiles were analyzed by Cellometer. EPA induced G1 phase cell cycle arrest in a dose-dependent manner. Treatment with 10 μM EPA increased G1 phase by 5.46% in SPEC2 cells (*p* = 0.0154). At 50 μM, G1 phase was increased by 6.72% in ARK-1 (*p* = 0.0139) and 10.42% in SPEC2 (*p* = 0.0003). Treatment with 100 μM EPA further elevated G1 phase to 13.11% in ARK-1 cells (*p* = 0.0002) (**A**). Western blotting results demonstrated that EPA inhibited CDK2, CDK4, and CyclinD1 expression in both cell lines after 24 h of treatment (**B**). For uncropped Western blot figures see Figure S2. * *p* < 0.05, ** *p* < 0.01 compared with control.

Given that EPA plays an important role in the regulatory function of cell cycle progression in both normal and cancer cells [25,26], image flow cytometry was utilized to assess whether EPA affects the progression of the cell cycle in USC cells. Both the ARK-1 and SPEC2 cells showed a dose-dependent increase in the percentage of cells in the G1 phase after 24 h of EPA treatment with a corresponding decrease in the percentage of cells in the S phase (Figure 3A). Treatment with 50 µM EPA increased the percentage of ARK-1 cells in the G1 phase by 6.72% and SPEC2 cells by 10.42% when compared to untreated cells. Consistent with these results, Western blotting confirmed that the expression levels of cyclin D1, CDK2, and CDK4 were markedly reduced in USC cells after EPA treatment for 24 h (Figure 3B). These findings imply that EPA suppresses the progression of the cell cycle by regulating critical factors implicated in the G1/S phase transition in USC cells.

### 3.3. EPA Triggers Cellular Stress Responses in USC Cells

**Figure 4:**
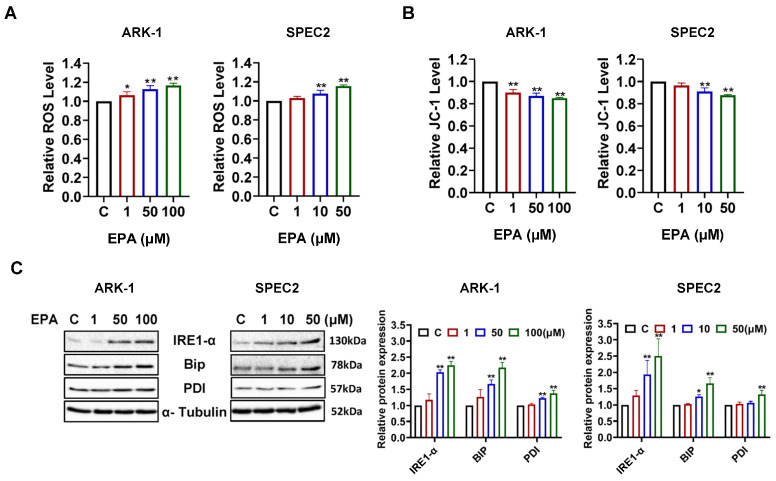
EPA increased cell stress in USC cells. After treatment of ARK-1 and SPEC2 cells with EPA for 3h, DCFH-DA assay showed that 1 μM EPA increased ROS by 6.33% in ARK-1 (*p* = 0.0246), 10 μM by 7.62% in SPEC2 (*p* = 0.0006), 50 μM by 12.58% in ARK-1 (*p* = 0.0002) and 15.59% in SPEC2 (*p* < 0.0001), and 100 μM by 16.68% in ARK-1 (*p* < 0.0001) (**A**). JC-1 assay indicated that EPA reduced mitochondrial membrane potential, with 1 μM decreasing it by 10.09% in ARK-1 (*p* = 0.0006), 10 μM by 9.07% in SPEC2 (*p* = 0.0017), 50 μM by 13.08% in ARK-1 (*p* = 0.0001) and 12.45% in SPEC2 (*p* = 0.0002), and 100 μM by 15.09% in ARK-1 (*p* < 0.0001) (**B**). Western blotting analysis confirmed that EPA induced the expression of IRE1α, Bip, and PDI in ARK1 and SPEC2 cells (**C**). For uncropped Western blot figures see Figure S3. * *p* < 0.05, ** *p* < 0.01 compared with control.

Considering that EPA plays a multifaceted regulatory role in cellular stress pathways in both normal and cancer cells [27,28,29], the impact of EPA on ROS and mitochondrial membrane potential was investigated. ROS analysis showed that EPA dramatically increased intracellular ROS levels in a dose-dependent fashion after treating ARK-1 and SPEC2 cells with different doses of EPA for 3 h (Figure 4A). Compared with untreated control cells, 50 µM EPA significantly increased ROS levels in ARK1 cells by 12.58% and SPEC2 cells by 15.59% respectively (*p* < 0.01). Results of JC-1 staining were consistent with an increased cellular stress response and showed a corresponding decrease in mitochondrial membrane potential, with 50 µM EPA decreasing membrane potential by 13.08% in ARK-1 and 12.45% in SPEC2 cells (*p* < 0.01), respectively (Figure 4B). In parallel, Western blotting revealed that 6–8 h EPA exposure upregulated the endoplasmic reticulum stress–related proteins BiP, PDI, and IRE1α (Figure 4C). Together, our results suggest that EPA causes oxidative and ER stress in USC cells.

### 3.4. EPA Induces Apoptosis in USC Cells

**Figure 5:**
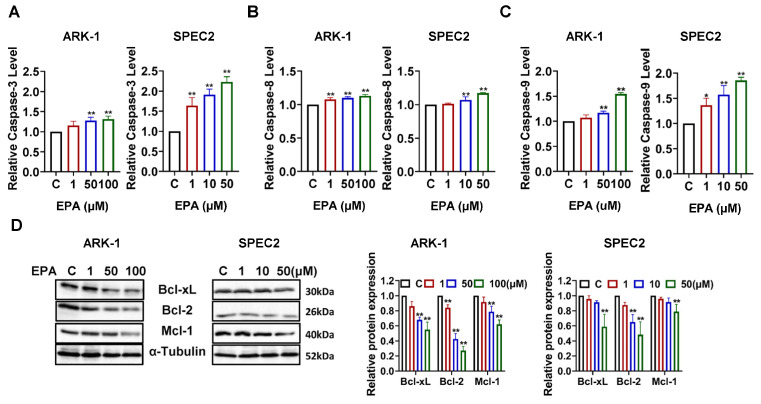
EPA induced apoptosis in USC cells. The ARK-1 cell line was treated with 1, 50, and 100 μM EPA for 4 h, and the SPEC2 cell line was treated with 1, 10, and 50 μM EPA for 4 h. The cleaved caspase levels were then detected by ELISA assay. The results showed that EPA promoted apoptosis in ARK-1 and SPEC2 cells by activating caspase pathways in a dose-dependent manner. Cleaved caspase-3 activity was increased by 63.86% and 91.08% in SPEC2 with 1 μM and 10 μM EPA, respectively (*p* < 0.0001), and by 27.45% in ARK-1 and 122.76% in SPEC2 at 50 μM (*p* = 0.0010 and *p* < 0.0001), and by 30.78% in ARK-1 at 100 μM (*p* = 0.0004). Cleaved caspase-8 activity was elevated by 7.74% in ARK-1 at 1 μM (*p* = 0.0003), 6.88% in SPEC2 at 10 μM (*p* = 0.0053), 9.95% in ARK-1 and 17.08% in SPEC2 at 50 μM (*p* < 0.0001), and 12.67% in ARK-1 at 100 μM (*p* < 0.0001). Cleaved caspase-9 increased by 36.35% and 57.52% in SPEC2 at 1 μM and 10 μM (*p* = 0.0138 and *p* = 0.0009), 16.73% in ARK-1 and 85.84% in SPEC2 at 50 μM (*p* = 0.0011 and *p* < 0.0001), and 54.51% in ARK-1 at 100 μM (*p* < 0.0001) (**A**–**C**). Western blotting results showed that after treating the two cell lines with different doses of EPA for 6–8 h, the expression of Bcl-xl, Bcl-2 and Mcl-1 was reduced (**D**). For uncropped Western blot figures see Figure S4. * *p* < 0.05, ** *p* < 0.01 compared with control.

Given that cellular stress and cell cycle arrest often serve as upstream triggers for apoptosis [30], we examined the potential of EPA to cause apoptosis in ARK-1 and SPEC2 cells. ELISA assays revealed a dose-dependent increase in activated cleaved caspase-3 after 4 h of EPA treatment, with 50 µM EPA increasing cleaved caspase-3 activity by 27.45% in ARK-1 and by 122.76% in SPEC2 cells (*p* < 0.01) (Figure 5A). EPA also enhanced the cleavage activity of caspase-8 and caspase-9. Treatment with 50 µM EPA increased the cleavage activity of caspase-8 and -9 in ARK-1 cells by 9.95% and 16.73% (*p* < 0.01), respectively, and increased the cleavage activity of caspase-8 and -9 in SPEC2 cells by 17.08% and 85.84% (*p* < 0.01), respectively, suggesting that both exogenous and endogenous apoptosis pathways were activated (Figure 5B,C). Western blotting revealed that after 8 h of exposure to EPA, expression levels of Bcl-xL, Mcl-1, and Bcl-2 in both cell types were significantly reduced (Figure 5D). These results indicate that EPA promotes apoptosis in USC cells by activating both the intrinsic and the extrinsic apoptotic pathways.

### 3.5. EPA Decreases the Invasive Capacity in USC Cells

**Figure 6:**
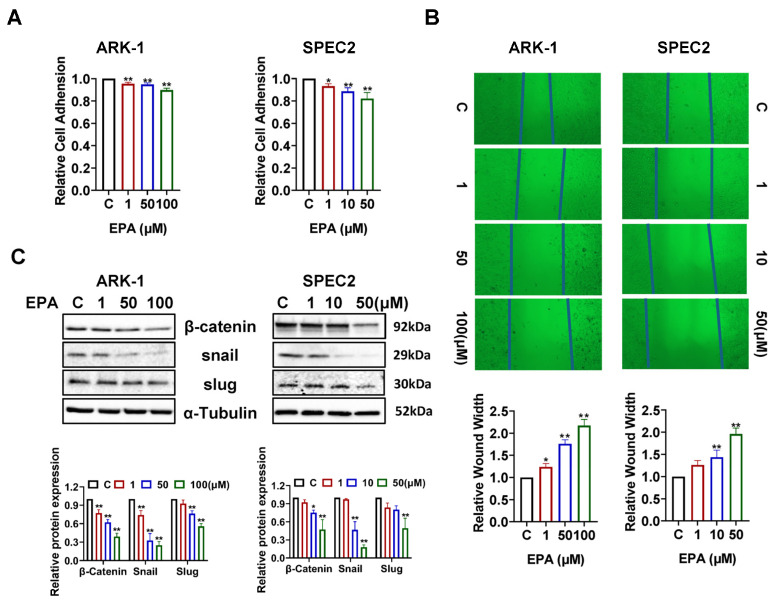
EPA inhibited cell adhesion and invasion in USC cells. EPA reduced cell adhesion to laminin-1 in ARK-1 and SPEC2 cells in a dose-dependent manner. In ARK-1, 50 and 100 μM EPA decreased adhesion by 5.17% (*p* = 0.0035), and 10.25% (*p* < 0.0001), respectively. In SPEC2, 1 μM, 10 μM, and 50 μM EPA reduced adhesion by 6.81% (*p* = 0.0370), 11.47% (*p* = 0.0012), and 17.85% (*p* < 0.0001), respectively. Assays were repeated three times (**A**). Wound healing assay revealed that EPA decreased cell migration in both cell lines (**B**). Western blotting results showed that EPA treatment decreased the expression of β-catenin, Slug, and Snail in both cell lines after 24 h of treatment (**C**). For uncropped Western blot figures see Figure S5. * *p* < 0.05, ** *p* < 0.01 compared with control.

EPA has shown anti-invasive properties in pre-clinical cancer models [31,32]. To explore the impact of EPA on cell–matrix interactions and cellular motility, adhesion and wound healing assays were conducted. Exposure to 50 µM EPA for 90 min significantly impaired cell adhesive ability, causing a 5.17% reduction in ARK-1 cells and a 17.85% reduction in SPEC2 cells (*p* < 0.01) (Figure 6A). Wound healing assays revealed that EPA suppressed cellular migration in a dose-dependent fashion. Specifically, treatment with 50 µM EPA increased wound healing width by 1.8-fold and 1.9-fold in ARK-1 and SPEC2 cells (Figure 6B). Additionally, Western blot assays demonstrated that exposing the cells to EPA for 24 h reduced epithelial–mesenchymal transition (EMT) by decreasing Slug, Snail, and β-catenin expression in ARK-1 and SPEC2 cell lines (Figure 6C). The results of our study indicate that EPA impairs cell adhesion and migration, possibly by influencing signaling pathways involved in EMT.

### 3.6. EPA Downregulates COX-2 Expression via NF-κB Signaling

**Figure 7:**
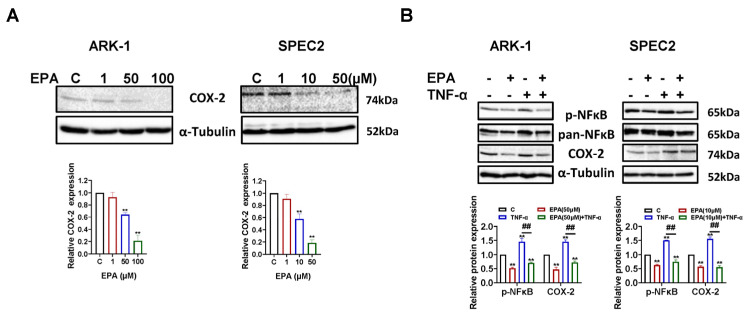
EPA inhibits the expression of COX-2 in USC cells. EPA treatment for 12 h effectively reduced the expression of COX-2 in the ARK-1 and SPEC2 cells, as shown by Western blotting analysis (**A**). The ARK-1 and SPEC2 were cultured in 10% FBS for 24 h and incubated with 0.5% charcoal-stripped FBS medium overnight. EPA effectively reduced the phosphorylation levels of NF-κB and COX-2 induced by TNF-α in both cell lines (**B**). For uncropped Western blot figures see Figure S6. ** *p* < 0.01.

As EPA exerts a potent anti-inflammatory effect by modulating various cellular signaling pathways [33], the impact of EPA on the cyclooxygenase-2 (COX-2) signaling in USC cells was examined. Western blot assays demonstrated that after 12 h of EPA administration, ARK-1 and SPEC2 cells displayed dose-dependent decreases in COX-2 protein levels (Figure 7A). To examine how the NF-κB pathway influences the decrease in COX-2 expression induced by EPA, ARK-1 and SPEC2 cells were exposed to TNF-α, EPA, or both together for 2 h. EPA effectively reduced TNF-induced phosphorylation levels of NF-κB and COX-2 (Figure 7B). These findings indicate that EPA reduces inflammation in USC cells by suppressing the NF-κB/COX-2 signaling pathway.

### 3.7. EPA Synergistically Enhances the Sensitivity of USC Cells to Carboplatin

**Figure 8:**
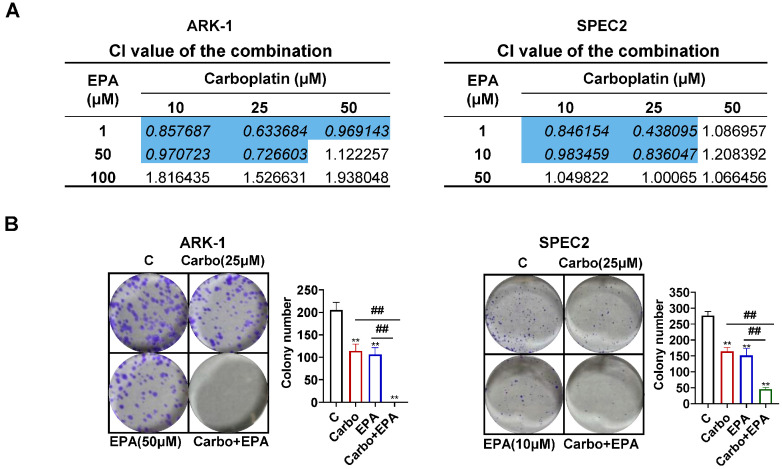

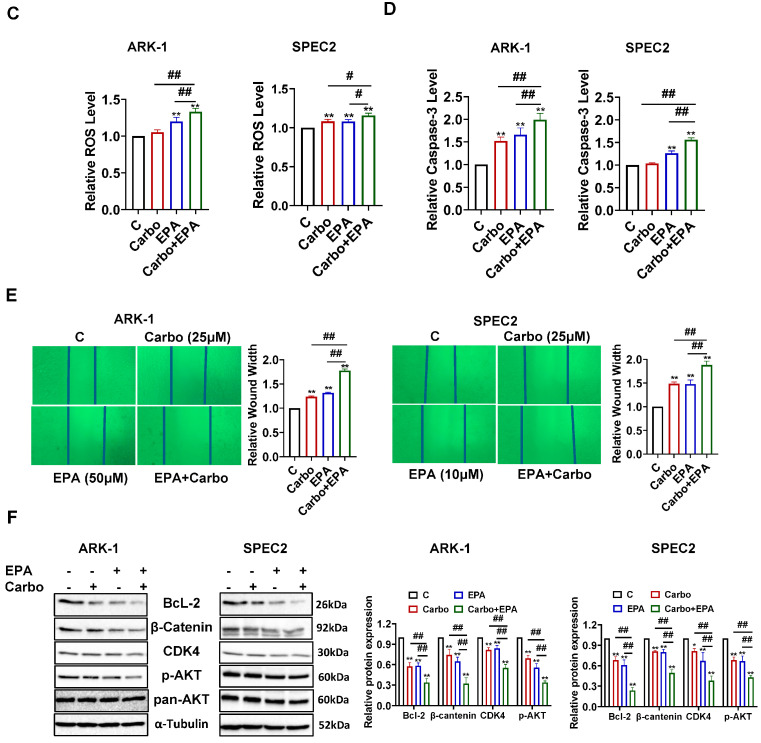
EPA synergistically enhances the sensitivity of USC cells to carboplatin. The ARK-1 and SPEC2 cells were incubated with specified doses of EPA, carboplatin, and their combinations for 72 h. The combination index was calculated based on the inhibition of cell growth (**A**). The combined treatment produced a stronger inhibitory effect on the colony-forming ability of both cell types (**B**) and induced higher levels of intracellular ROS (**C**) and cleaved caspase 3 (**D**). Combination treatment with EPA and carboplatin had a greater impact on cellular migration compared with each agent alone (**E**). Western blot assay was used to analyze the effect of EPA, carboplatin and the combination on phos-AKT, PDI, BcL-2, β-catenin and CDK4 after 24 h of treatment in both cell lines (**F**). For uncropped Western blot figures see Figure S7. * *p* < 0.05, ** *p* < 0.01 compared with control.

Because platinum-based chemotherapy is a crucial component of the treatment paradigm used in the treatment of USC [1,5], we explored whether EPA could enhance the responsiveness of USC cells to carboplatin. ARK-1 and SPEC2 cells were exposed to EPA, carboplatin, or a combination of both for 72 h. MTT assays demonstrated a synergistic interaction between EPA and carboplatin as evidenced by a combination index less than one. Notably, the synergistic effect was most prominent at lower dose combinations. For example, 1 µM EPA plus 25 µM carboplatin yielded a CI of 0.63 in ARK-1 cells (Figure 8A). Treatment with 50 µM EPA in ARK-1, 10 µM in SPEC2 and 25 µM carboplatin each reduced colony growth by roughly 50%, while their combination nearly abolished colony formation compared to monotherapy (>99% reduction; Figure 8B). The combined treatment also resulted in larger ROS accumulation compared to EPA or carboplatin alone (*p* < 0.05) (Figure 8C). In addition, co-administration of 50 µM EPA in ARK-1 cells and 10µM in SPEC2 cells in combination with carboplatin (25 µM) produced a greater increase in cleavage caspase-3 activity in both cell lines compared to treatment with each agent alone (*p* < 0.01) (Figure 8D). The wound healing experiments showed that combination treatment dramatically decreased cell migration compared to EPA or carboplatin alone. EPA increased wound healing width by 1.30-fold in ARK-1 cells and migration by 1.52-fold in SPEC2 cells, while carboplatin increased ARK-1 and SPEC2 cell wound healing width by 1.25-fold and 1.51-fold, respectively. The combination treatment effectively reduced cell migration, increasing ARK-1 cell wound healing by 1.79-fold and SPEC2 cell migration by 1.92-fold (*p* < 0.01) (Figure 8E). Western blotting results demonstrated that the combination treatment led to a more significant effect on the expression of BcL-2, β-Catenin, CDK4 and phosphorylated AKT in both cell lines compared with the single drug treatment (Figure 8F). Collectively, these findings point to a synergistic effect of combined treatment in suppressing cell proliferation, increasing cellular stress and apoptosis, and decreasing invasive potential in USC cells.

## 4. Discussion

This study is the first to comprehensively demonstrate that EPA, an omega-3 PUFA predominantly found in marine sources, exerts potent anti-tumorigenic effects in USC, an aggressive form of endometrial cancer with limited treatment options and a poor overall prognosis. Our findings show that EPA significantly inhibits proliferation, colony formation, adhesion, migration, the inflammatory response, and the AKT/mTOR signaling pathway in the ARK-1 and SPEC2 cells. Simultaneously, EPA induces cellular stress, induces G1 phase cell cycle arrest, triggers apoptosis, and activates the MAPK pathway. Notably, the EPA–carboplatin combination significantly decreased cell growth, colony formation, and cell migration while also inducing cell stress and apoptosis, suggesting a potential synergy effect between EPA and carboplatin in USC cells compared to single agent treatment. These findings confirm that EPA is a promising bioactive fatty acid with anti-proliferative and anti-invasive properties in USC cells.

EPA inhibits cell proliferation by disrupting key checkpoints in the cell cycle, causing cancer cells to arrest in the G1 or G2 phase [34]. EPA-induced cell cycle arrest in the G1 or G2 phase is most likely related to the inhibition of PI3K/mTOR signaling pathways and the regulation of MAPK and ERK signaling pathways. Inhibition of Akt phosphorylation by EPA results in downregulation of cyclin D1 expression and reduced CDK4/6 activity, thereby blocking the G1-to-S phase transition in cancer cells [25,26]. Likewise, the influence of EPA on the activity of the MAPK signaling pathway markedly changed the regulation of the cell cycle as mediated by cyclin D1, thereby impacting the progression of the G1 phase [25,35]. Consistent with the role of EPA in other cancer types, our findings demonstrate that EPA significantly leads to G1 phase arrest, accompanied by downregulation of cyclin D1, CDK2 and CDK4, phosphorylated AKT and phosphorylated S6, and increased expression of phosphorylated ERK1/2 in USC cells. These results support the hypothesis that modulation of cell cycle checkpoints, such as cyclin D1 and PI3K/mTOR and MAPK signaling pathways, is a key mechanism for EPA-induced arrest of the cell cycle and suppression of cell viability in USC cells.

EPA exerts profound modulatory effects on several cellular stress pathways, including oxidative stress, the endoplasmic reticulum (ER) stress/unfolded protein response (UPR), and mitochondrial function [36,37,38]. In addition, EPA exhibits antioxidant properties in some cancer types and normal cells [39,40]. For instance, 48 h treatment with EPA in HepG2 cells decreased ROS levels by at least 40% and enhanced antioxidant capacity by about 50–70%, likely due to enhanced mitochondrial membrane potential and increased mitochondrial biogenesis [39]. It is generally accepted that cell stress induced by EPA suppresses cancer cell proliferation by disrupting cellular homeostasis, lowering the apoptosis threshold, stimulating activation of caspase and the degradation of poly (ADP-ribose) polymerase (PARP) [29,36,38]. Our research revealed that EPA substantially increased intracellular ROS levels, decreased mitochondrial membrane potential, and stimulated PDI, IRE1α, and Bip expression in USC cells. These molecular alterations coincide with the activation of caspase-3, -8, and -9 and a decrease in the expression of the anti-apoptotic proteins Bcl-xL, Mcl-1, and Bcl-2, suggesting the participation of both intrinsic and extrinsic apoptotic pathways.

By controlling key signaling pathways and processes involved in extracellular matrix remodeling, EPA has been shown to decrease the invasive potential of cancer cells [41,42]. EPA inhibited the directed migration of the esophageal squamous cell carcinoma line TE-1 and suppressed macrophage-induced cell migration by attenuating the expression of matrix metalloproteinases in gastric cancer cells [31,41]. EPA treatment effectively counteracts inflammation-induced MMP-9 upregulation and inhibits NOTCH1 activity, thereby reducing cellular invasiveness in colon cancer cells [43]. EPA treatment effectively lowered their capacity for adhesion and invasion and altered the expression of EMT-related proteins, such as β-catenin, Slug, and Snail, in both USC cells lines. Based on previous results and our findings, we believe that EPA is a strong bioactive agent that can inhibit cell invasion by modulating EMT processes, reducing extracellular matrix degradation, and attenuating motility signals in USC cells.

Chronic inflammation promotes tumor progression by sustaining growth signals, promoting angiogenesis and invasion, and weakening anti-tumor immunity, largely through increased COX-2, prostaglandin E2, TNF-α, and IL-6 [44,45]. Pre-clinical investigations have found that EPA modulates these pathways by decreasing the synthesis of pro-inflammatory eicosanoids and cytokines, suppressing NF-κB and COX-2 signaling pathways, and altering membrane phospholipid composition, thereby disrupting inflammatory signaling cascades across various cancer types [46,47,48]. This study has confirmed that EPA reduces COX-2 expression and inhibits the expression of COX-2 and phosphorylated NF-κB induced by TNF-α in ARK-1 and SPEC2 cells. These findings substantiate the potential role of EPA in mitigating tumor-associated inflammation in USC cells.

Studies have demonstrated that EPA can improve the therapeutic efficacy of conventional chemotherapeutic agents by augmenting the susceptibility of tumor cells to drug-induced cytotoxicity through synergistic pathways [36,38,49]. Mechanistically, EPA modulates membrane lipid composition, increases oxidative stress, inhibits inflammatory responses and angiogenesis, and suppresses pro-survival signaling pathways including PI3K/Akt and NF-κB, thereby lowering the apoptosis threshold and increasing the vulnerability of tumor cells to cytotoxic effects induced by DNA-damaging agents, such as platinum-based compounds, and anti-metabolites, including 5-fluorouracil [36,49]. In glioblastoma cells, EPA enhanced the suppressive effects of temozolomide by altering lipid metabolism, increasing ROS production, disrupting glucose and lactate metabolism, and suppressing Akt and GSK3β activity [50]. Results from this research demonstrate that EPA can synergistically enhance the inhibitory effects of carboplatin on cell growth and colony formation by enhancing cellular stress and activating caspase-mediated apoptosis. Notably, the combined treatment substantially enhances its inhibition on USC cell migration compared to single-drug treatment. The synergistic interaction of EPA and carboplatin provides pre-clinical evidence for another potential therapeutic strategy for highly invasive and recurrent USC, a cancer with limited treatment options.

## 5. Conclusions

Circulating EPA typically comprises approximately 0.5–2% of total plasma fatty acids, corresponding to an estimated concentration of 10–50 µM under basal conditions, depending on dietary intake, population characteristics, health status, and measurement methodology. These levels can increase substantially with oral supplementation, reaching approximately 300–900 µM, with even higher concentrations observed under pharmacological dosing. In the present study, the highest concentration of EPA used (100 µM) falls within the range of total plasma EPA achievable under oral supplemented conditions, supporting the physiological relevance of our findings. Our results show that EPA exerts multifaceted anti-tumorigenic activity in USC cells, including reduced proliferation and invasiveness, induction of G1 cell cycle arrest, activation of stress and apoptotic responses, and attenuation of pro-inflammatory signaling. Moreover, the combined administration of EPA and carboplatin appears to potentiate growth inhibition and anti-invasive effects, implying possible synergistic interaction. Consistent with our previous findings demonstrating that DHA exerts anti-proliferative and anti-invasive effects in preclinical models of ovarian cancer, the present study extends these observations to USC and further provides new mechanistic insight into the anti-cancer effects of EPA. Overall, our findings lay the groundwork for future preclinical and clinical evaluation of EPA as a promising adjunct therapeutic strategy for USC.

## Data Availability

All data generated or analyzed during this study are included in this article. The datasets used and/or analyzed during the current study are available from the corresponding authors upon reasonable request.

## Supplementary Materials

The following supporting information can be downloaded at: https://www.mdpi.com/article/10.3390/cancers18071120/s1, Figure S1: Uncropped Western blot figures of Figure 2C; Figure S2: Uncropped Western blot figures of Figure 2B; Figure S3: Uncropped Western blot figures of Figure 4C; Figure S4: Uncropped Western blot figures of Figure 5D; Figure S5: Uncropped Western blot figures of Figure 6C; Figure S6: Uncropped Western blot figures of Figure 7; Figure S7: Uncropped Western blot figures of Figure 8F.

## Abbreviations

The following abbreviations are used in this manuscript:

BiP	heavy chain binding protein
CDK	cyclin/cyclin-dependent kinases
COX-2	cyclooxygenase-2
DCFH-CA	2’,7’-dichlorofluorescin diacetate
DHA	docosahexaenoic acid
DMSO	dimethyl sulfoxide
EMT	epithelial–mesenchymal transition
EPA	eicosapentaenoic acid
ER	endoplasmic reticulum
FBS	fetal bovine serum
IL-6	interleukin-6
IC_50_	half-maximal inhibitory concentration
IRE1-α	inositol-requiring protein 1
MAPK	mitogen-activated protein kinase
MTT	methylthiazolyldiphenyl-tetrazolium bromide
mTOR	mammalian target of rapamycin
NF-κB	nuclear factor kappaB
PA	palmitic acid
PARP	poly(ADP-ribose) polymerase
PBS	phosphate buffer saline
PI	propidium iodide
PI3K	phosphoinositide 3-kinase
PUFA	polyunsaturated fatty acid
ROS	reactive oxygen species
TNF-α	tumor necrosis factor alpha
UPR	unfolded protein response
USC	uterine serous carcinoma
