# Supplementary figures and images for: Eicosapentaenoic Acid Suppresses Tumor Growth and Enhances Chemosensitivity via AKT/mTOR Signaling in Uterine Serous Carcinoma
Source: Cancers (Basel). 2026 Mar 31;18(7):1120. doi: 10.3390/cancers18071120 (PMC13072313; doi:10.3390/cancers18071120)

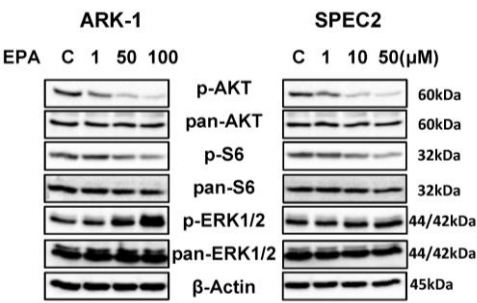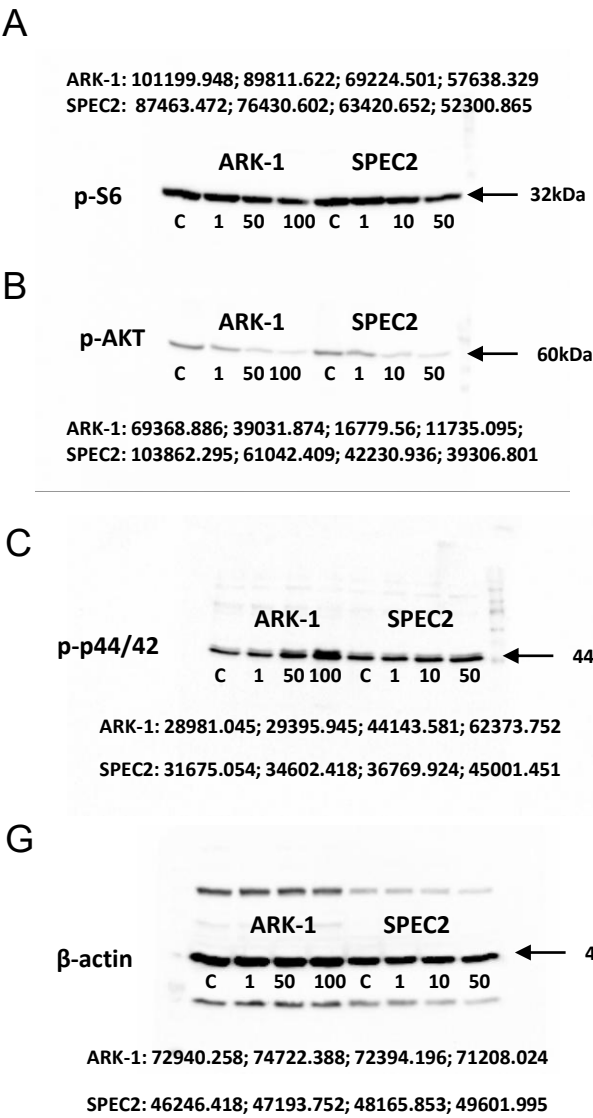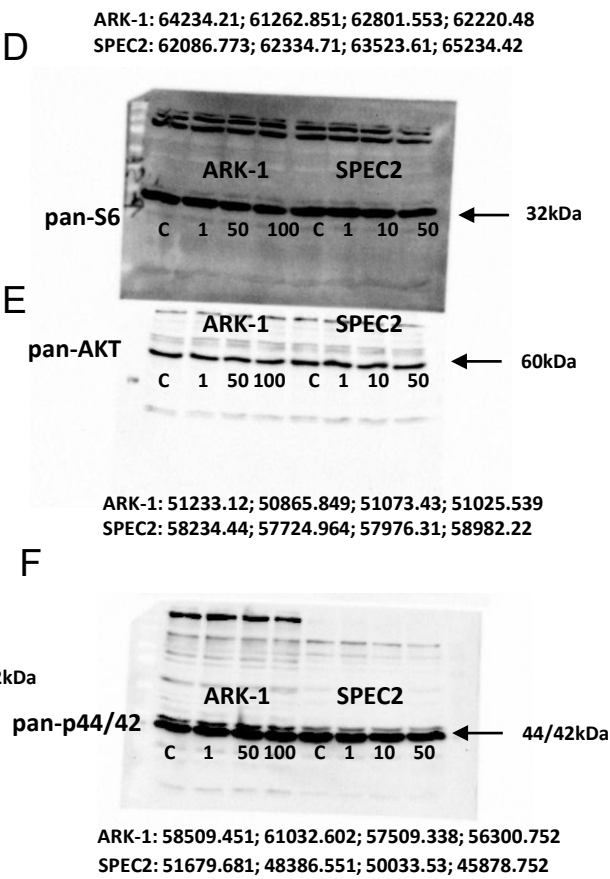

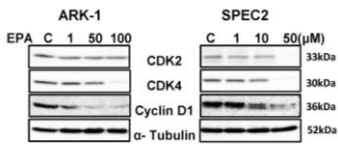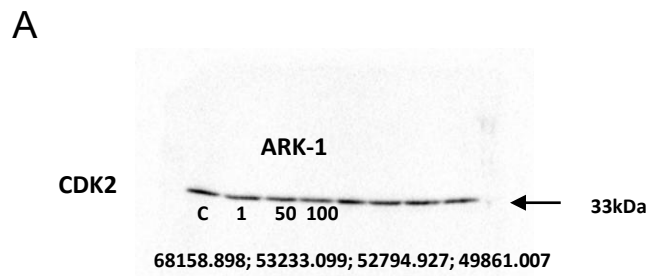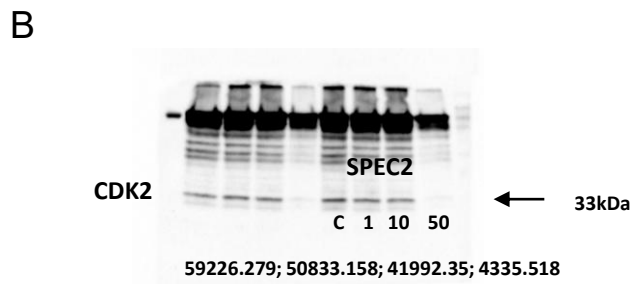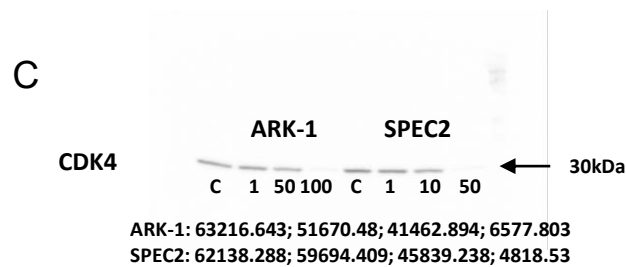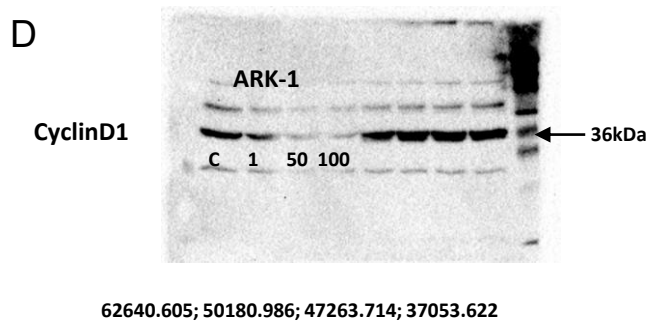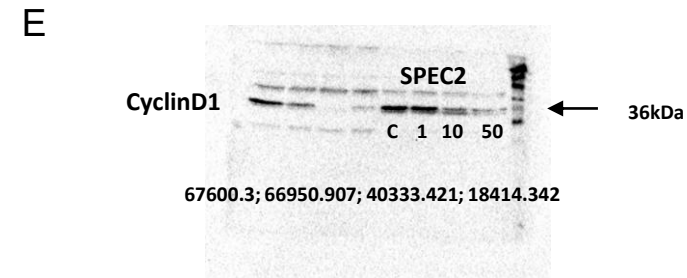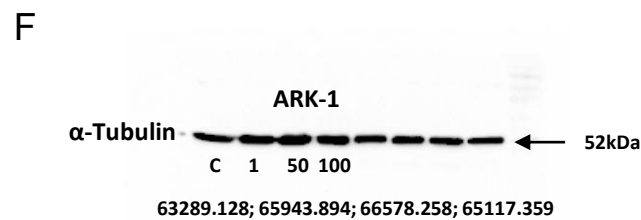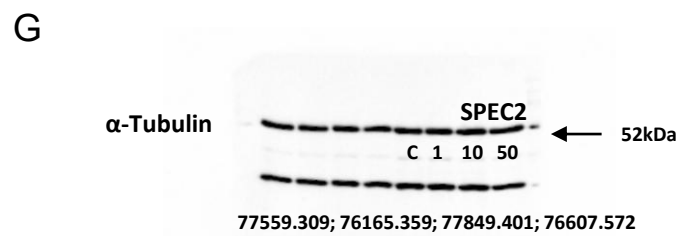

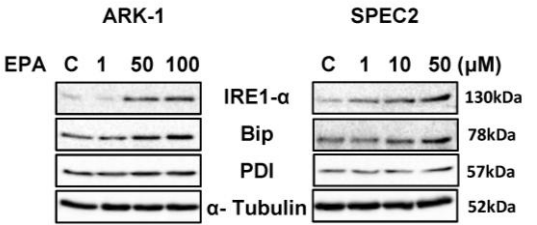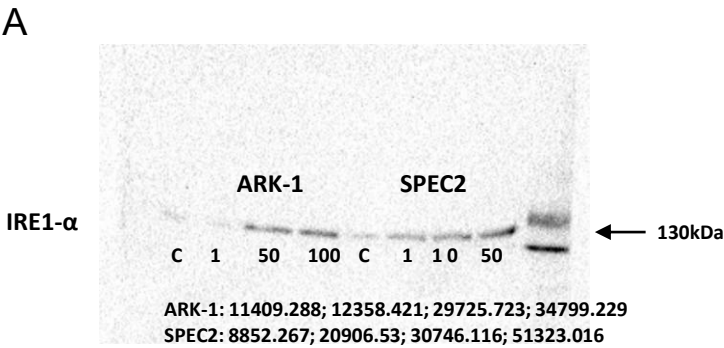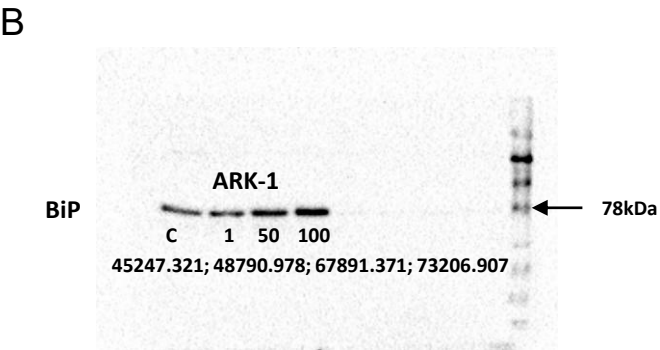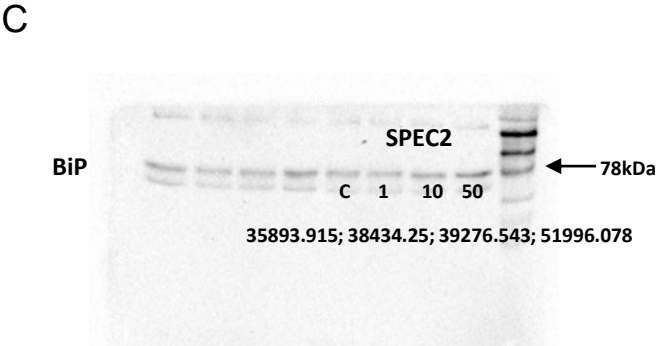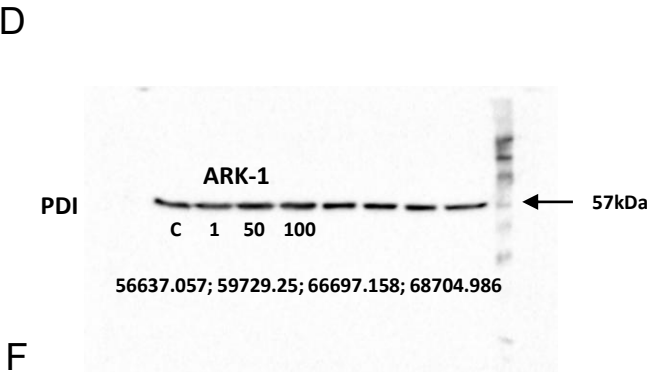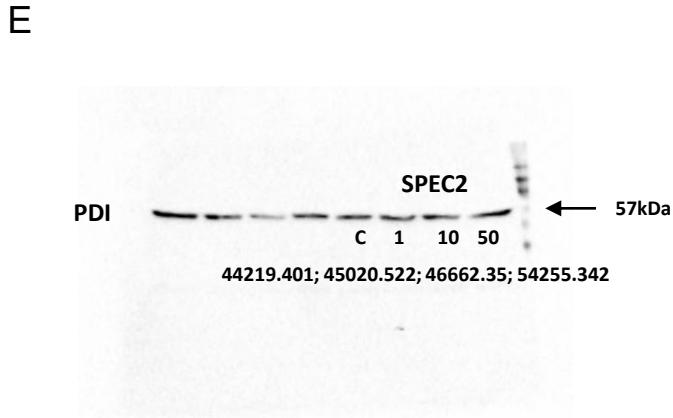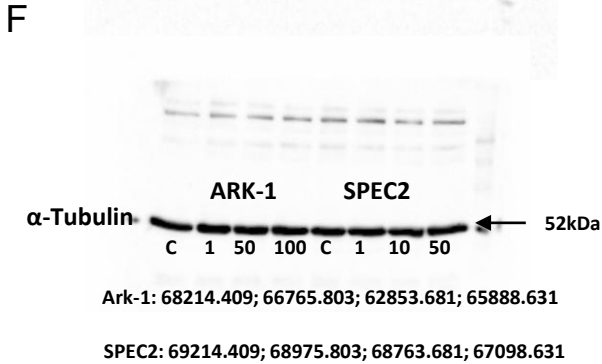

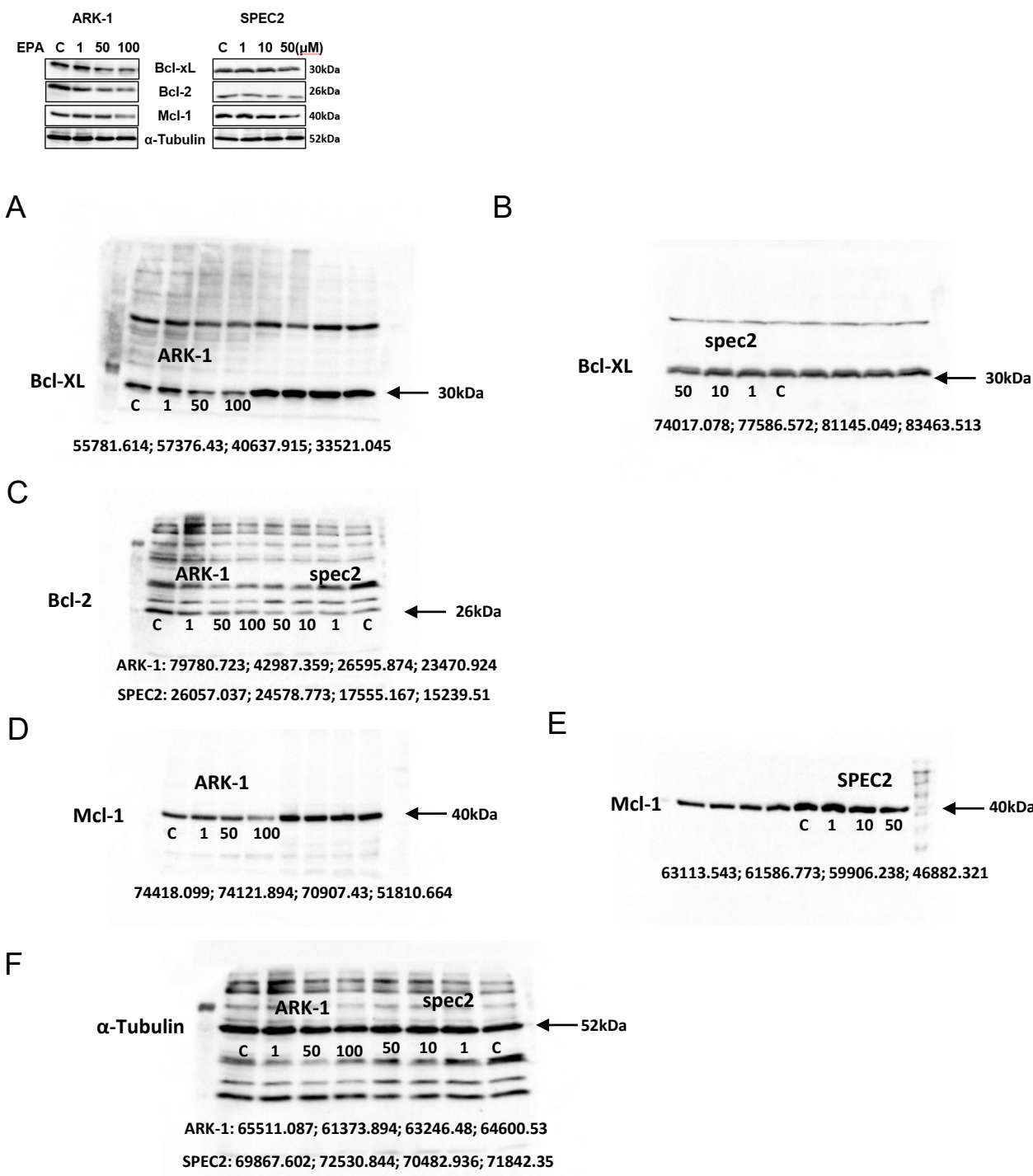

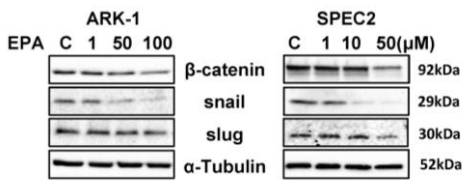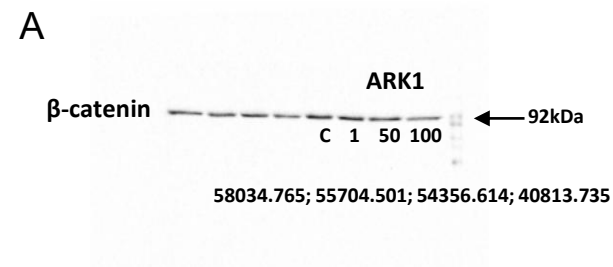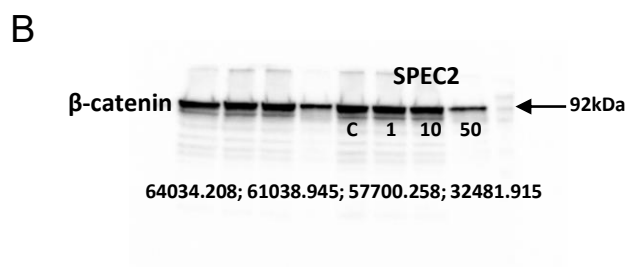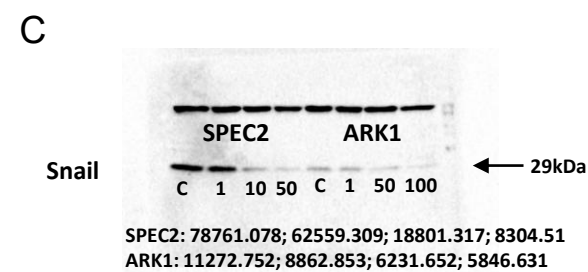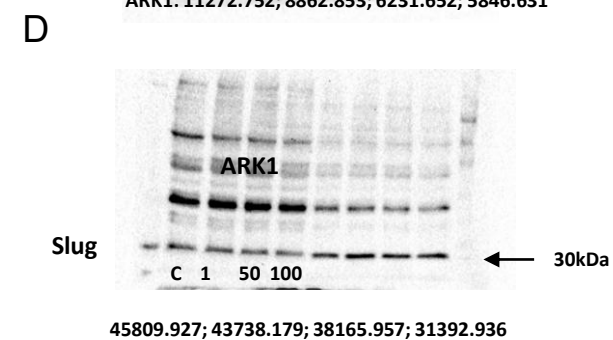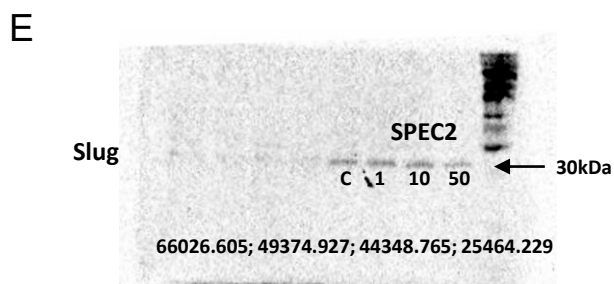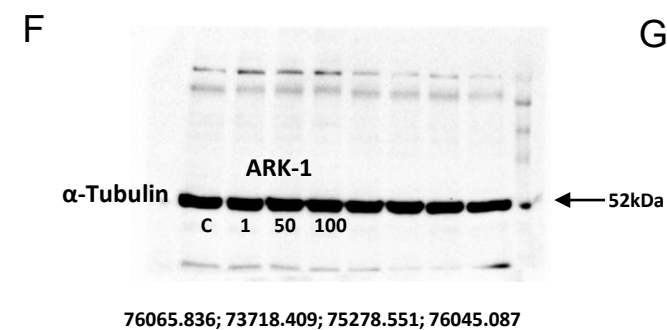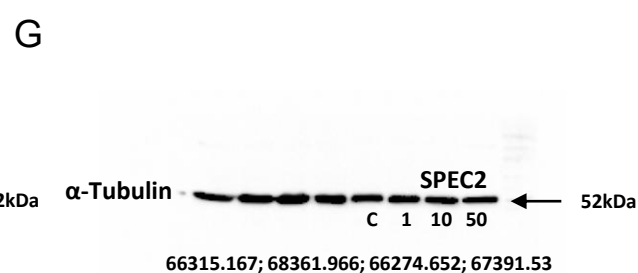

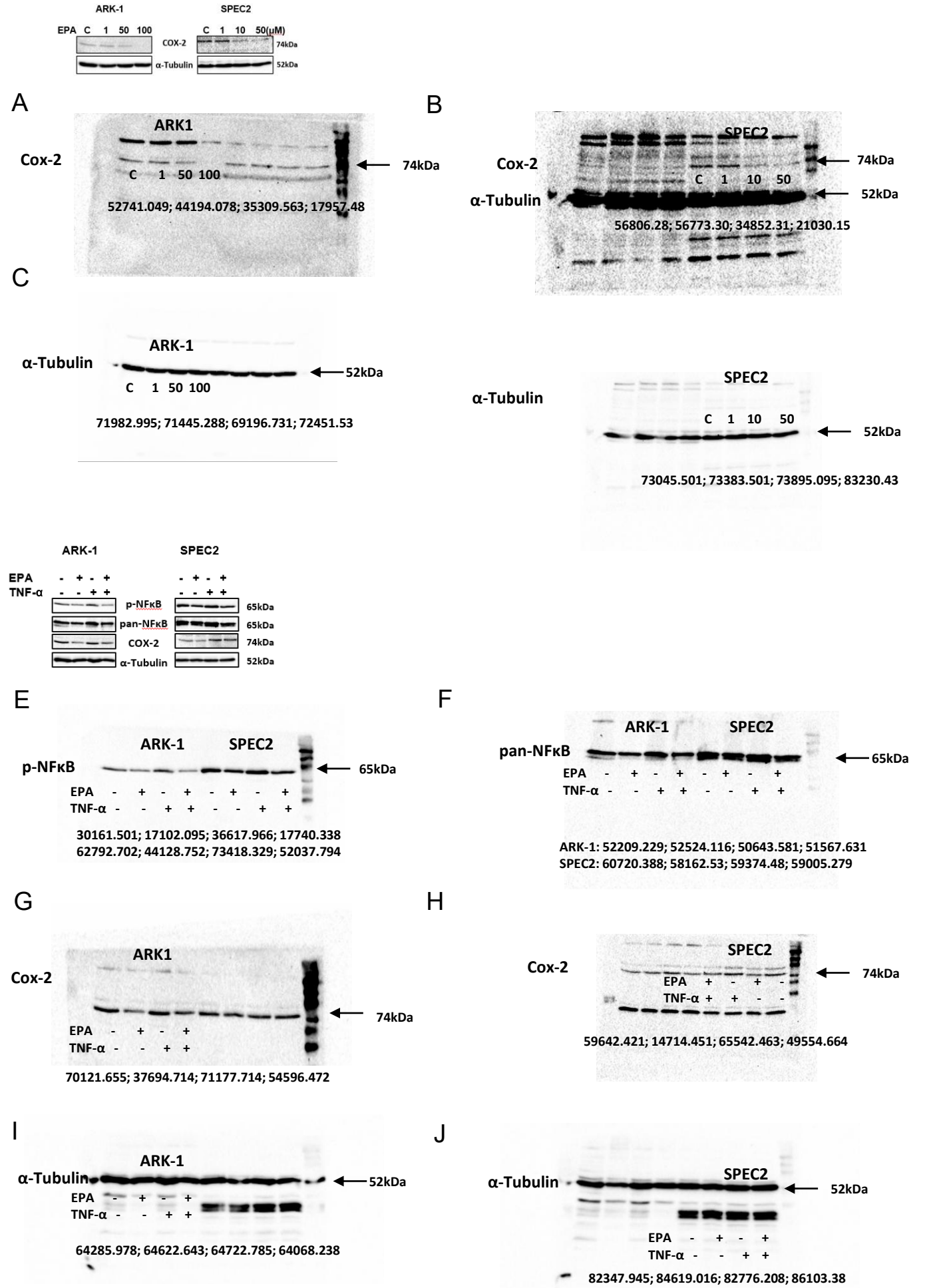

S7

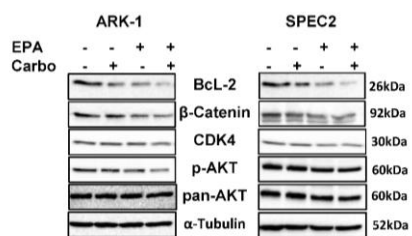

B

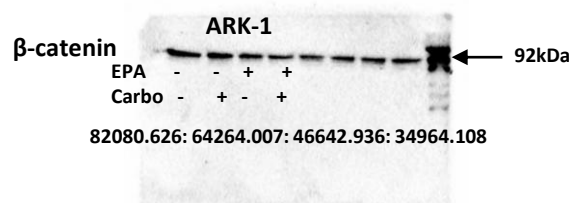

A

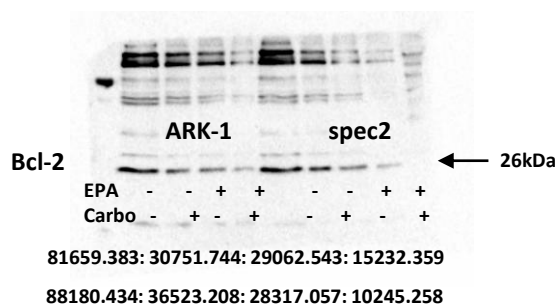

C

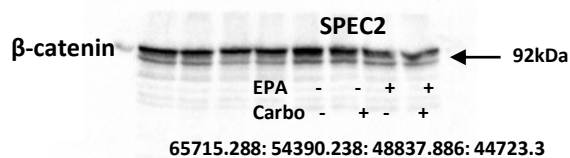

D

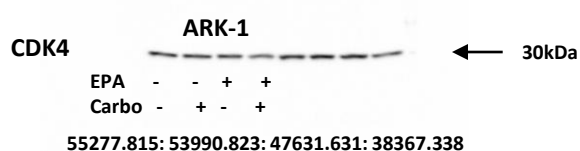

E

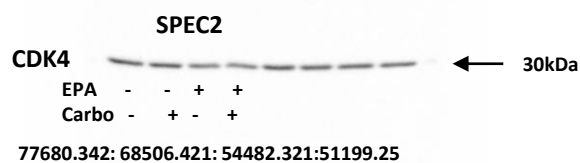

F

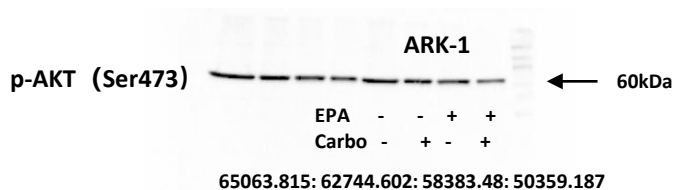

G

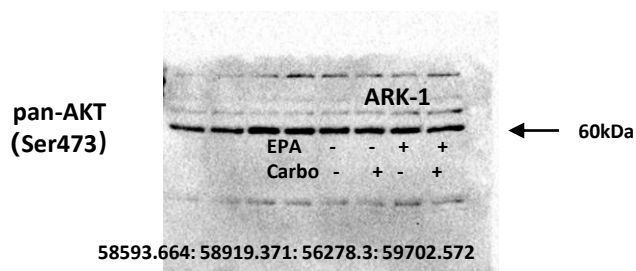

H

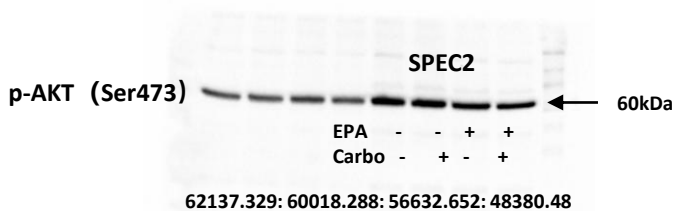

I

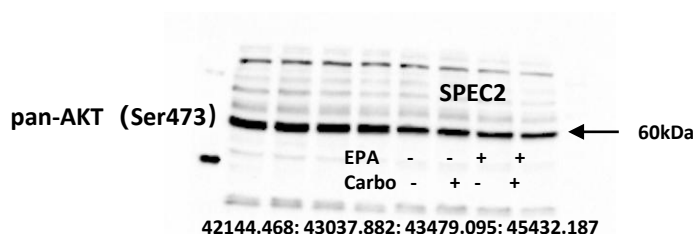

J

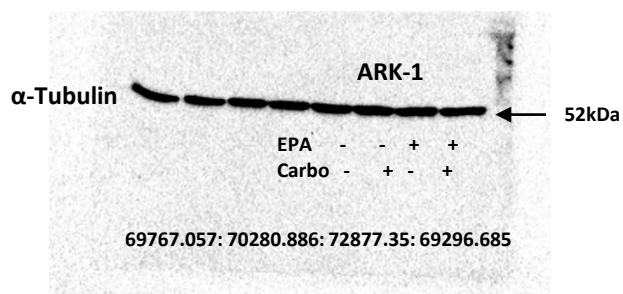

K

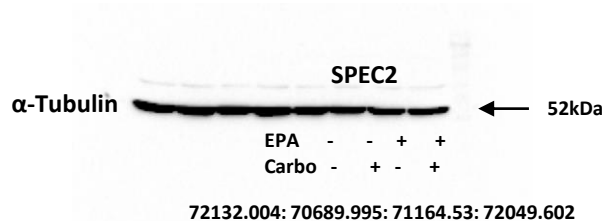

Supplement: Supplementary file 1 [file cancers-18-01120-s001.zip › cancers-4213678-supplementary.pdf]
